# Prenatal Diagnosis of Cleft Lip and Palate: A Retrospective Study

**DOI:** 10.3390/jcm13164804

**Published:** 2024-08-15

**Authors:** Anca Daniela Brăila, Constantin Marian Damian, Cristina-Crenguţa Albu, Oana Botoacă, Laurențiu Mihai Dȋră, Ştefan-Dimitrie Albu, Matei Georgian Brăila, Andreea-Mariana Bănățeanu, Cristian-Viorel Poalelungi, Claudia Florina Bogdan-Andreescu

**Affiliations:** 1 Department of Obstetrics and Gynecology, University of Medicine and Pharmacy of Craiova, 200349 Craiova, Romania; anca.braila@umfcv.ro (A.D.B.); constantin.damian@umfcv.ro (C.M.D.); laurentiu.dira@umfcv.ro (L.M.D.); 2 Department of Genetics, Faculty of Dentistry, “Carol Davila” University of Medicine and Pharmacy, 020021 Bucharest, Romania; 3 Department of Speciality Disciplines, Faculty of Dental Medicine, “Titu Maiorescu” University, 031593 Bucharest, Romania; andreea.banateanu@prof.utm.ro (A.-M.B.); claudia.andreescu@prof.utm.ro (C.F.B.-A.); 4 Department of Periodontology, Faculty of Dentistry, “Carol Davila” University of Medicine and Pharmacy, 020021 Bucharest, Romania; stefan-dimitrie.albu@drd.umfcd.ro; 5 Faculty of Medicine, “Carol Davila” University of Medicine and Pharmacy, 020021 Bucharest, Romania; matei-georgian.braila2022@stud.umfcd.ro; 6 Department of Obstetrics and Gynecology, Faculty of Medicine, “Carol Davila” University of Medicine and Pharmacy, 020021 Bucharest, Romania; cristian.poalelungi@umfcd.ro

**Keywords:** cleft lip and palate, prenatal diagnosis, 3D ultrasound imaging, genetic tests

## Abstract

Cleft lip and/or palate are prevalent congenital anomalies. Early and accurate diagnosis allows proper case management. **The Objective**: This retrospective cohort study aimed to investigate the association between cleft lip and palate and other congenital anomalies. **Methods**: This study analyzed 17 pregnancies prenatally diagnosed with cleft lip and palate. The investigations consisted of ultrasound examination, fetal karyotyping through amniocentesis, and family tree analysis. In the presence of an abnormal fetal karyotype, the parental karyotype was also indicated. **Results**: Of the 17 cases identified, 9 (52.94%) were syndromic and 8 (47.06%) were non-syndromic. The genetic syndromes identified in association with cleft lip and palate in this study included translocation syndrome (one case), Patau syndrome, trisomy 13 (seven cases), and Edwards syndrome, mosaic trisomy 18 (one case). **Conclusions**: A comprehensive approach ensures a thorough assessment and accurate diagnosis. Early detection and a multidisciplinary approach allow appropriate case management.

## 1. Introduction

Cleft lip and/or palate (CLP) is one of the most prevalent congenital anomalies of the craniofacial region. This condition not only alters physical appearance but also significantly affects the functionality of the oral and nasal structures [1,2]. Understanding embryological development and the resulting pathophysiological changes due to CLP is important for diagnosis.

During the first 10 weeks of embryogenesis, facial development involves the growth and fusion of several distinct processes. The primary palate forms the initial portion of the mouth, while the secondary palate subsequently develops to complete the formation of hard and soft palates. These structures are supposed to fuse at the midline of the nasal septum. However, in cases of CLP, fusion is incomplete [3].

The failure of fusion can be partial or complete, leading to various manifestations:

- Cleft lip (CL): involves only the lip, generally the upper lip, and may range from a small notch to a complete separation extending into the nose.
- Cleft lip and palate (CLP): involves both the lip and the palate. This is a more severe manifestation that significantly affects speech and eating, and is often associated with other dental and orthodontic issues.
- Cleft palate (CP): affects only the palate, either hard, soft, or both. This type primarily impacts speech and swallowing.

Orofacial clefts are among the most common head and neck birth defects worldwide. According to Geneser et al., the incidence of orofacial clefts is approximately 1 in 700 live births [4]. However, this rate exhibits considerable geographic variation, suggesting the influence of genetic, environmental, and possibly socioeconomic factors [4].

A comprehensive systematic review reported prevalence rates of 0.33 per 1000 live births for cleft lips and 0.45 per 1000 for cleft lip and palate combinations [5].

The clinical classification of orofacial clefts includes syndromic and non-syndromic forms, based on associated anomalies and/or medical conditions [6]. This classification guides both clinical management and genetic counseling. Syndromic CLPs are characterized by additional anomalies or medical conditions that go beyond the cleft itself. These clefts are part of broader genetic syndromes and are often associated with other developmental, structural, and/or cognitive anomalies. Syndromic CLPs account for approximately 30% of all cases of cleft lip and palate cases [7].

The genetic basis for many syndromic CLPs follows Mendelian inheritance patterns (autosomal dominant, autosomal recessive, or X-linked). These conditions exhibit significant variability in how they manifest (expressivity), and not all individuals who carry a predisposing genetic variant will exhibit the trait (incomplete penetrance) [7]. This genetic complexity necessitates thorough clinical and genetic evaluation to identify the specific syndrome and tailor management accordingly.

Non-syndromic CLPs, the more common form, do not have associated anomalies and constitute approximately 70% of all CLP cases [8]. These clefts are considered to have a complex etiology, involving interactions between multiple genetic factors and environmental influences. The absence of additional congenital anomalies often leads to a primary focus on the cleft for surgical and supportive care.

CLPs can be diagnosed prenatally or postnatally during neonatal examination. Prenatal diagnosis typically involves ultrasound scans conducted in the second trimester of pregnancy, at around 20 weeks of gestation, which have become a routine component of antenatal care in most European countries [9]. At this stage of gestation, the face is sufficiently developed to allow the visualization of an orofacial cleft via ultrasound [10]. However, the success rate of detecting CLP through ultrasound largely depends on different factors such as gestational age, maternal obesity, volume of amniotic fluid, coexisting fetal anomalies, fetal position and mobility, as well as the expertise of the sonographers [11,12].

CLP-associated conditions pose unique challenges for care providers due to their complexity and the various associated complications that may arise. In such cases, genetic tests are decisive because they can identify specific mutations and chromosomal abnormalities associated with CLPs.

Fetuses with associated anomalies, regardless of the presence of aneuploidy, have a poor prognosis, with a very high mortality rate, and an increased likelihood of prematurity and a low birth weight. Fetuses with isolated CLPs have a good prognosis, with a gestational age and weight at birth comparable with term babies [13].

While prenatal ultrasound can detect early CPLs, amniocentesis is advised to evaluate associated genetic conditions, as part of prenatal diagnosis [14,15]. Prenatal identification of CLP enables detailed parental counseling and early intervention. This process includes discussions regarding the potential implications of the anomaly and the prognosis for surgical correction. Furthermore, given that CLPs may co-occur with other anomalies and genetic abnormalities, early detection via prenatal screening allows for an extensive fetal examination to identify additional, potentially more severe anomalies.

This study aimed to investigate the association between CLPs and other congenital anomalies.

## 2. Materials and Methods

This study retrospectively analyzed a cohort of pregnancies examined in a private clinic in Bucharest between 1 May 2021 and 1 April 2024. The inclusion criteria were pregnancies diagnosed with oral clefts.

During this period, 3347 pregnant women were examined. Following specialized investigations, 30 pregnancies were prenatally diagnosed with various congenital fetal malformations located in the head and neck region. From this group, we isolated a study cohort comprising 17 pregnancies diagnosed prenatally with different types of orofacial clefts.

The standard protocol in cases of CLP consists of the following:

- Advanced ultrasound imaging using 3D/4D imaging technology provides detailed views of the fetal anatomy, allowing for the precise assessment of detected anomalies.
- Genetic testing: amniocentesis is commonly performed to obtain a sample of amniotic fluid for fetal karyotyping. This procedure helps to identify chromosomal abnormalities.
- Parental karyotyping and family tree: when an abnormal fetal karyotype is identified, parental karyotyping is recommended to determine if the chromosomal abnormality is inherited or de novo. This information is crucial for genetic counseling and future pregnancy planning.

The ultrasound examination was conducted transabdominally using a Voluson E10 BT18 ultrasound machine (GE Healthcare division, Wauwatosa, WI, USA), equipped with an RM6C three-dimensional/four-dimensional (3D/4D) volumetric probe. This advanced imaging technology allowed detailed visualization of fetal structures.

The following classification was used for clefts:

- Unilateral cleft lip (UCL), left (UCLL), and right (UCLR);
- Bilateral cleft lip (BCL);
- Cleft palate (CP);
- Cleft palate with unilateral cleft lip (UCLP), left (UCLPL), and right (UCLPR);
- Bilateral cleft lip and palate (BCLP).

Upon identification of the oral clefts during the ultrasound scan, further investigations were recommended. These included amniocentesis, which involves sampling the amniotic fluid, and the determination of the fetal karyotype to check for any genetic abnormalities.

In the presence of an abnormal fetal karyotype, the parental karyotype was indicated from peripheral blood. This additional step helps to identify any genetic anomalies that might be inherited and provides valuable information for genetic counseling and future pregnancy planning.

Patients were provided with a clear explanation of the consent form, detailing the clinical, paraclinical, and ultrasound examinations. The procedural steps necessary for conducting specialized investigations were also explained, along with the content of the consent form regarding the protection of personal data.

Ethics approval was obtained from the local Ethics Committee of the Alco San Medical Center in Bucharest, Romania. Written informed consent for participation was obtained from all the parents.

## 3. Results

The results are summarized in Table 1. The percentage of CLPs in this study was 0.51% during the pregnancy screening conducted in the second trimester at the clinical center. Of the 17 cases identified, 9 (52.94%) were syndromic and 8 (47.06%) were non-syndromic cases of CLP. The genetic syndromes identified in association with CLP in this study included translocation syndrome (one case male), Patau syndrome, trisomy 13 (seven cases, five male and two female)*,* and Edwards syndrome, mosaic trisomy 18 (one case male). All cases were isolated and non-hereditary, based on paternal karyotype and family tree analysis.

Among these 17 pregnancies with CLPs, 15 were associated with other anomalies, and abnormal chromosomal formulas were identified in 9 (52.94%) cases. The frequency of associated anomalies is detailed in Table 2.

The CLP types are presented in Table 3. There was only one case (5.88%) with bilateral CLP, while the remaining cases were unilateral: seven (41.18%) on the right side and nine (52.94%) on the left side.

**Figure 1:**
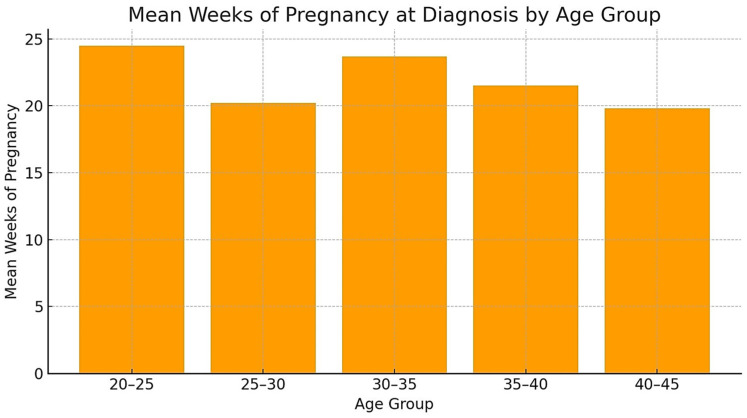
Mean weeks of pregnancy at diagnosis according to age.

**Figure 2:**
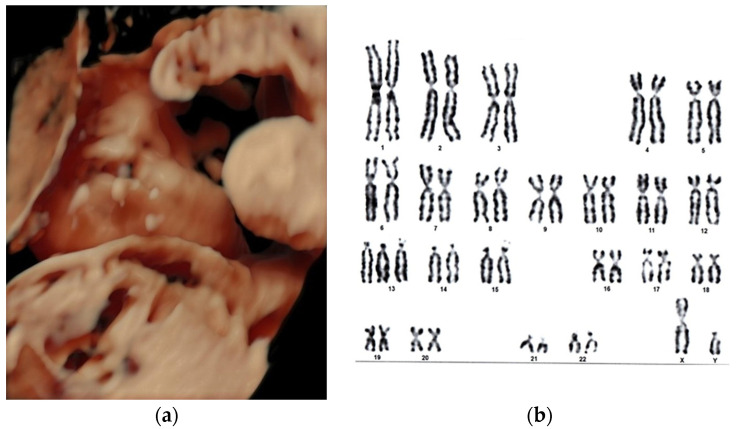
Unilateral cleft labial diagnosed prenatally at 19 weeks of pregnancy and confirmed at 23 weeks, isolated, sporadic, syndrome, non-hereditary case, Patau syndrome (trisomy 13): 47, XY, +13. (**a**) Three-dimensional ultrasound evaluation of fetal viscerocranium indicated unilateral left cheiloschisis, prenatally diagnosed at 19 w and confirmed at 23 w of pregnancy; (**b**) Fetal karyotype from amniotic cell culture: 47, XY, +13.

**Figure 3:**
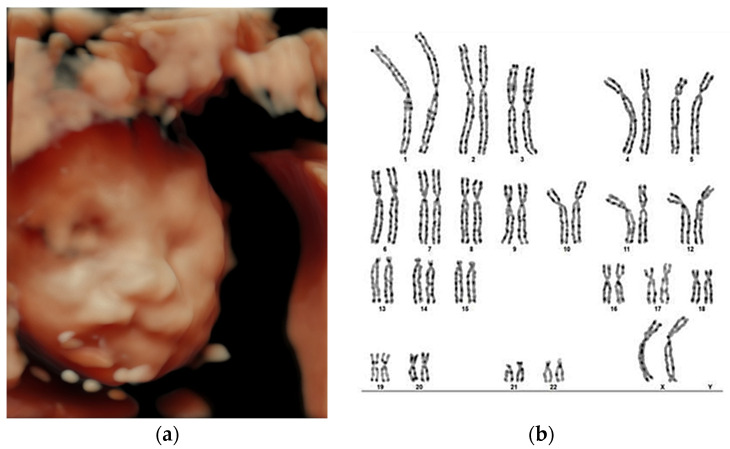
Unilateral cleft labial diagnosed prenatally at 23 weeks of pregnancy, isolated case, sporadic, non-syndromic, non-hereditary, confirmed postnatally. (**a**) Three-dimensional ultrasound evaluation of fetal profile indicated unilateral cheiloschisis, prenatally diagnosed at 23 weeks of pregnancy; (**b**) Fetal karyotype from amniotic cell culture: 46, XX.

Figure 1 shows mean weeks of pregnancy at diagnosis by age group indicating differences in the timing of diagnosis, and Figure 2 and Figure 3 illustrate two representative cases, one syndromic and one non-syndromic.

The mean maternal age was 29.94 years, and the mean gestational age at diagnosis was 21.17 weeks. Table 4 presents the association between maternal age and the occurrence of CLP, distinguishing between syndromic and non-syndromic cases.

The mean paternal age was 36.18 years. Table 5 presents the association between paternal age and the occurrence of CLP, distinguishing between syndromic and non-syndromic cases.

All pregnancies were monofetal, with 5 (29.41%) female and 12 (70.59%) male fetuses. Most pregnancies were first pregnancies (13, or 76.47%), while 4 (23.52%) were second pregnancies.

## 4. Discussion

Ultrasound screening is a non-invasive method, aimed to assess fetal anatomy and detect any fetal anomalies [16], with a high degree of accuracy, to determine types of facial clefts [17]. Three-dimensional ultrasound imaging enables reconstruction, allows for more precise visualization of fetal dental and craniofacial structures [18], and improves the prenatal detection rate of CLPs, especially those involving the secondary palate [12,19,20].

The reported rate of prenatal CLP detection in the current literature varies, depending on the database used. For instance, 62 fetuses of 38,760 pregnancies in the Netherlands were identified with CLPs, and 39% of cases had associated anomalies in a prospective national study using 2D ultrasound [21].

The high incidence of CLP in our study, 17 cases from 3347 pregnancies, with 3 (17.65%) cases being second opinions, reflects our status as a leading referral center for prenatal diagnosis and comprehensive multidisciplinary care. Our advanced diagnostic capabilities enable early detection and detailed assessment of craniofacial anomalies, attracting a significant number of referrals from neighboring regions. Our multidisciplinary team, which includes specialists in obstetrics–gynecology, genetics, pediatrics, dentistry, and psychology, collaborates to provide detailed genetic counseling. This helps families understand the potential genetic factors and recurrence risks associated with CLPs and develop individualized treatment plans. This integrated approach ensures optimal outcomes in the management of these congenital conditions and offers psychosocial support for affected families.

All diagnoses resulting from ultrasound prenatal examination were confirmed by the final diagnoses obtained from postnatal physical examination or fetal autopsy. As a result, high-precision ultrasonography with 3D imaging reconstruction had 100% accuracy.

In our study, the most frequent type of cleft was UCLP, with four cases being non-syndromic and eight cases being syndromic. Additionally, the only case of BCLP observed was syndromic.

In a related study based on data from a prenatal diagnosis center, 15 cases of 2944 pregnant women examined in the first trimester were described, with all cases confirmed during second-trimester ultrasonography [22].

Accurate prenatal diagnosis allows for early intervention planning and better management of the condition. The prognosis of a newborn diagnosed with CLP significantly depends on the accuracy of the prenatal diagnosis and the presence of associated malformations [23]. Additionally, the prognosis is influenced by whether the CLP is an isolated anomaly or part of a syndrome involving other organs or systems.

A comprehensive epidemiologic study by Calzolari et al., which analyzed nearly 6 million births in Europe, identified that musculoskeletal, cardiovascular, and central nervous system defects are frequently associated with cleft lip and/or palate [24].

Another study from India identified the following anomalies associated with CLP: facial region anomalies were the most frequent (21%), followed by ocular anomalies (17%), central nervous system anomalies (15%), gastrointestinal system anomalies (3%), and urogenital system anomalies (2%) [14]. Additionally, 34 patients (2%) had recognized non-chromosomal syndromes [25].

A high incidence of additional anomalies was recorded in a large cohort study from Germany [17]. The most common anomalies associated with CLP were cardiac, cerebral, extremity, facial, and ocular [17].

In our study, the most frequent defects associated with CLP were cardiovascular, anomalies affecting craniofacial bones and limbs, and brain anomalies. Other malformations appeared in the kidneys, eyes, spine, and stomach.

Although increased nuchal translucency is considered a marker for the presence of CLP [10], we founded this association in just one case.

Of the 17 pregnancies, only 2 cases (11.76%) had isolated CL, while the remaining 15 cases (88.24%) had associated anomalies. Specifically, 52.94% of the cases had chromosomal anomalies. The two cases without associated anomalies, confirmed postnatally, involved female fetuses.

The chromosomal syndromes recognized in this study were, in order of frequency, Patau syndrome or trisomy 13 (seven cases), translocation syndrome (one case) and Edwards syndrome, mosaic trisomy 18 (one case), all with CLP. The most frequent aneuploidy in our study was Patau syndrome, which is recognized by the following triad: microphthalmia, polydactyly, and CP [18]

The majority of chromosomal syndromes in this study were found in male pregnancies, with seven out of nine cases involving male fetuses. There were two cases of female pregnancy with Patau syndrome (trisomy 13).

The number of trisomy 13 (Patau syndrome) cases in this study is notably high compared to the estimated incidence, which ranges from 1 in 10,000 to 20,000 births [26].

The majority of Patau syndrome cases occurred in primiparous women, with only one case occurring in a second pregnancy highlighting a possible area for focused prenatal monitoring and research. No placental abnormalities were detected in the cases of trisomy 13, despite such abnormalities being commonly associated with this condition [27].

Increased maternal age is a known risk factor for chromosomal aneuploidies, including trisomy 13 [28,29]. In this study, the mean maternal age for cases with Patau syndrome was 28.29 years. Only one case involved a maternal age over 35 years, suggesting that while maternal age is a risk factor, trisomy 13 can occur in younger mothers as well.

Paternal age has been linked to an increased risk of offspring inheriting certain disorders [30], including several genetic diseases [31,32]. However, in our study, which included two cases of paternal age over 50, only one aneuploidy was identified. Due to the small sample size, we could not establish a strong association between fathers over 50 and CLPs. This finding aligns with a recent study that reported a positive association between advanced paternal age and Down syndrome, while finding a negative association with CL with or without CP [33].

In a large cohort study, chromosomal abnormalities were detected in 62 out of 168 cases with CLP [17], with the most common being trisomy 13, followed by trisomy 18. Other chromosomal abnormalities included Cri-du-chat syndrome, trisomy 21, trisomy 14, ectrodactyly–ectodermal dysplasia–clefting (EEC), Turner syndrome, and Klinefelter syndrome [17].

With new advances in genetic testing, around 500 syndromes associated with CLPs have been identified [34]. Van der Woude syndrome is the most common genetic syndrome associated with CPLs [35]. Other syndromes with clefting as a significant feature include ectrodactyly–ectodermal dysplasia–clefting syndrome [36], blepharocheilodontic syndrome [37], and Patau syndrome [38].

Research since the 2000s has identified numerous genetic syndromes associated with CLPs: Edwards syndrome [39], Stickler syndrome [40], Holzgreve syndrome, Marfan syndrome, myotonic dystrophy, Klippel-Feil syndrome, Potter sequence [41], Down syndrome [42,43], Ellis-van Creveld syndrome [44], Hartsfield syndrome [45], and others.

Comprehensive prenatal genetic tests have become an essential part of prenatal screening and diagnosis in cases where congenital anomalies are present. Screening tests are conducted from the mother’s blood sample and diagnostic tests involve invasively obtaining fetal tissue via chorionic villus sampling and amniocentesis and have over 99.9 percent accuracy [46]. Traditionally, this testing has been performed through fluorescence in situ hybridization, karyotyping, or chromosomal microarray analysis. New methods, such as genomic hybridization, have been developed with similar accuracy [47]. However, karyotype may still be the most efficient and cost-effective method in certain clinical scenarios where aneuploidy is suspected [48].

Amniocentesis is commonly used in Romania to obtain amniotic fluid for prenatal diagnosis. While highly valuable for detecting chromosomal abnormalities, it is crucial to consider the associated risks, particularly the risk of miscarriage.

This risk is relatively low [49,50], but when counseling parents and obtaining their consent for amniocentesis it is a significant issue that must be discussed as a procedure-related complication.

Recent review studies have demonstrated that the risks associated with amniocentesis, particularly when used for karyotyping, are low [51,52], due to advances in ultrasound-guided techniques, even when performed later in pregnancy, after 24 weeks [53,54,55].

Diagnostic genetic test options include karyotyping, fluorescence in situ hybridization, chromosomal microarray, methylation studies, targeted testing, targeted gene panels, exome sequencing, and genome sequencing. The indication for a particular test depends on the findings from prenatal imaging, the results of genetic screening tests, and family history analysis. In our study, karyotyping was indicated due to its high accuracy and the suspicion of aneuploidy. This method facilitated the precise identification of chromosomal abnormalities linked to CLPs, offering critical data for genetic counseling and enabling timely, well-informed decisions on pregnancy management.

The survival rate for individuals with CLP varies significantly depending on whether the condition is isolated or associated with genetic or chromosomal syndromes. According to Goldrick et al., survival rates are higher in cases of isolated CLP, while the prognosis is poorer for CLP cases linked to chromosomal abnormalities [56]. Following genetic counseling, patients often choose to terminate pregnancies when CLP is associated with severe fetal chromosomal abnormalities. In this context, 15 women opted for pregnancy termination after prenatal ultrasound detected multiple and severe congenital malformations, whether isolated or in combination with severe chromosomal abnormalities. Ethical considerations play a crucial role in these decisions, reflecting the complex interplay between medical information, familial impact, and patient autonomy in prenatal care.

All cases of non-syndromic CLP in this study are non-familial and occur as isolated congenital anomalies. These findings are confirmed by different studies [57] and have a multifactorial etiology involving both genetic and environmental factors.

The etiology of non-syndromic CLP remains largely unknown, though mutations in candidate genes have been identified in a small proportion of cases based on a review of research from 2012 [58]. However, newer research has identified pathogenic gene variants in the etiology of CLP [59,60].

Furthermore, recent research indicated that subjects with CLP had the highest percentage of positive family history and that the condition predominantly affects male subjects [61,62]. Similarly, in our study clefts were more frequent in male than female fetuses.

Increased maternal age could not be correlated with syndromic cases in our study. The association between CLPs and maternal age remains controversial [63]. A meta-analysis indicated no correlation between maternal age and CLPs [64], while other studies have suggested the opposite [65,66,67]. In our study, the highest syndromic cases were in the 20–29 age group.

The average weeks of pregnancy at the time of diagnosis for each age group indicate that younger mothers tend to receive later diagnoses, while older mothers are diagnosed earlier on average. This discrepancy may be attributed to advanced maternal age being a risk factor for pregnancy complications, fetal aneuploidy, and genetic disorders [68], leading to more frequent and earlier prenatal screenings for older mothers.

Understanding the relative risk of CLP based on genetic background and environmental factors including nutrition, smoking, alcohol, medications, and chemicals, will be invaluable for genetic counseling and the development of future preventive measures [69].

Based on the results of this study, we can conclude that associated anomalies are common among pregnancies with CLP, highlighting the importance of genetic analysis. The search for additional malformations in the case of CLP and underlying genetic conditions is essential.

Early evaluation with genetic testing and management is recommended for families affected by oral clefts to help manage the emotional and psychological stress associated with this condition [8]. Counseling is a crucial component of family support.

Genetic analysis is a crucial component of prenatal care for these cases. This comprehensive approach—ultrasound scans with 3D imaging reconstruction, fetal karyotype analysis, and pedigree analysis—ensures thorough assessment and accurate diagnosis.

## 5. Suggestions for Future Research

Future research should prioritize evaluating the comparative effectiveness of advanced non-invasive prenatal testing (NIPT) against traditional invasive diagnostic methodologies in the early detection of cleft lip and palate (CLP). Such comparative analysis is crucial for optimizing prenatal screening protocols and improving clinical outcomes.

The potential role of paternal age in the incidence of CLP is another important area for further research. Future studies should aim to include larger and more diverse populations to explore the genetic mutations that could be associated with increased paternal age.

Additionally, exploring the interplay between genetic susceptibilities and environmental factors in the etiology of CLP is essential for identifying targeted preventative interventions.

Furthermore, there is a significant need for longitudinal cohort studies to monitor the long-term phenotypic developments and psychosocial consequences associated with CLP. These studies will enhance our understanding of the condition’s progression and facilitate management strategies and patient support systems.

This comprehensive approach will significantly contribute to the advancement of personalized medicine and improve prognostic outcomes for affected individuals.

## 6. Limitation of the Study

The current research is subject to several limitations. Specifically, in this retrospective study, the limitations include the small number of cases and the reliance on karyotyping as the sole genetic test. Karyotyping was chosen due to its accuracy and cost-effectiveness. Non-invasive genetic screening tests are more expensive than karyotyping.

## 7. Conclusions

Detecting CLPs during prenatal ultrasound screening is essential for informed decision-making. High-precision ultrasound and 3D imaging reconstruction allow early detection.

The presence of additional malformations is an essential parameter in the decision to perform an invasive genetic test, which establishes the diagnosis.

## Figures and Tables

**Table 1:** Summative table with subjects and data analyses.

No.	Maternal Age	Paternal Age	History	Ultrasonography	CLP	Weeks of Pregnancy	Fetal Karyotype	CLP Heterogenity Linked to Fetal Karyotype and Pedigree Analysis	Genetic Component of CLPs	Observation
1.	24	26	first pregnancy	female monofetal pregnancy	UCLR	31.3	46, XX	isolated, sporadic, non-hereditary case	non-syndromic case	second opinion confirmed postnatally
2.	26	30	first pregnancy	male monofetal pregnancy intrauterine growth restriction, left equinovarus	UCLPL	23.1	46 XY, t(7;16) (p14;p11.)	isolated, sporadic, syndromic, non-hereditary case	translocation syndrome	termination of pregnancy, confirmed by fetal autopsy
3.	31	51	first pregnancy	male monofetal pregnancy, nuchal translucency, spina bifida, and congenital heart anomaly with moderate regurgitation of the tricuspid valve	UCPLR	17	46, XY	isolated, sporadic, non-hereditary case	non-syndromic case	termination of pregnancy, confirmed by fetal autopsy
4.	26	32	first pregnancy	male monofetal pregnancy, oligohydramnios, hypoplastic nasal bone, bilateral polycystic kidney and intrauterine growth restriction	BCLP	20	46, XY/47, XY, +13	isolated, sporadic, syndromic, non-hereditary case	Patau syndrome, mosaic trisomy 13	termination of pregnancy, confirmed by fetal autopsy
5.	31	37	second pregnancy	male monofetal pregnancy, ventricular septal defect	UCLPR	20	47, XY, +13	isolated, sporadic, syndromic, non-hereditary case	Patau syndrome, trisomy 13	termination of pregnancy, confirmed by fetal autopsy
6.	29	27	first pregnancy	female monofetal pregnancy, ocular hypertelorism, polycystic kidney and polydactyly	UCLPR	25	46, XX/47, XX, +13	isolated, sporadic, syndromic, non-hereditary case	Patau syndrome, mosaic trisomy 13	termination of pregnancy confirmed by fetal autopsy
7.	39	45	first pregnancy	male monofetal pregnancy, hypoplastic nasal bone, agenesis of the corpus callosum and ventriculomegaly	UCLPL	19	46, XY	isolated, sporadic, non-hereditary case	non-syndromic case	termination of pregnancy confirmed by fetal autopsy
8.	31	34	first pregnancy	female monofetal	UCLR	31.6	46, XX	isolated, sporadic, non-hereditary case	non-syndromic case	second opinion, confirmed postnatally
9.	27	30	second pregnancy	female monofetal pregnancy, microcephaly	UCLPR	19	46, XX/47, XX, +13	isolated, sporadic, syndromic, non-hereditary case	Patau syndrome, mosaic trisomy 13	termination of pregnancy, confirmed by fetal autopsy
10.	29	33	second pregnancy	male monofetal pregnancy, equinovarus	UCLPR	20	46, XY	isolated, sporadic, non-hereditary case	non-syndromic case	termination of pregnancy, confirmed by fetal autopsy
11.	38	45	first pregnancy	male monofetal pregnancy, lax nuchal cord	UCLL	20	46, XY	isolated, sporadic, non-hereditary case	non-syndromic case	termination of pregnancy confirmed by fetal autopsy
12.	24	30	first pregnancy	female monofetal pregnancy, moderate gastric distension	UCLL	23	46, XX	isolated, sporadic, non-hereditary case	non-syndromic case	second opinion, confirmed postnatally
13.	36	42	first pregnancy	male monofetal pregnancy, ventriculomegaly	UCLPR	18	47, XY, +13	isolated, sporadic, syndromic, non-hereditary case	Patau syndrome, trisomy 13	termination of pregnancy confirmed by fetal autopsy
14.	26	32	first pregnancy	male monofetal pregnancy, ventriculomegaly	UCLPL	19	47, XY, +13	isolated, sporadic, syndromic, non-hereditary case	Patau syndrome, trisomy 13	termination of pregnancy confirmed by fetal autopsy
15.	41	50	second pregnancy	male monofetal pregnancy, retrognathia, moderate micrognathia, hemi ventriculomegaly, suspicion of lissencephaly	UCLPR	18	46, XY/47, XY, +18	isolated, sporadic, syndromic, non-hereditary case	Edwards syndrome, mosaic trisomy 18	termination of pregnancy confirmed by fetal autopsy
16.	28	40	first pregnancy	female monofetal pregnancy, coarctation of the aorta	UCLPL	17	46, XX	isolated, sporadic, non-hereditary case	non-syndromic case	termination of pregnancy confirmed by fetal autopsy
17	23	31	first pregnancy	male monofetal pregnancy, Spalding sign, double cranial contour, developmental arrest	UCLPL	19	47, XY, +13	isolated, sporadic, syndromic, non-hereditary case	Patau syndrome, trisomy 13	termination of pregnancy confirmed by fetal autopsy

Unilateral cleft lip right (UCLR), Cleft palate with unilateral cleft lip left (UCLPL), Cleft palate with unilateral cleft lip right (UCLPR), Bilateral cleft lip and palate (BCLP), Unilateral cleft lip left (UCLL).

**Table 2:** The association between CLPs and other anomalies.

Cases without Associated Anomalies	Bone Anomalies	Brain Anomalies	Cardio-Vascular Anomalies	Cranio-Facial Bones Anomalies	Digestive Anomalies	Limb Anomalies	Ocular Anomalies	Renal Anomalies	Intrauterine Growth Restriction	Other
2 (11.76%)	1 (5.88%)	3 (17.65%)	7 (41.18%)	4 (23.53%)	1 (5.88%)	3 (17.65%)	1 (5.88%)	2 (11.76%)	3 (17.65%)	3 (17.57%)
	spina bifida	agenesis of the corpus callosum, lissencephaly, microcephaly	coarctation of the aorta, regurgitation of the tricuspid valve, ventriculomegaly, ventricular septal defect	hypoplastic nasal bone, retrognathia with micrognathia, Spalding sign	moderate gastric distension	equinovarus, polydactyly	hypertelorism	polycystic kidney		lax nuchal cord, oligohydramnios, nuchal translucency

**Table 3:** Classification of CLPs.

Type of CLPs	Non-Syndromic Cases	Syndromic Cases	Total
Unilateral cleft lip (UCL)	4 (23.52%)	0 (0.00%)	4 (23.52%)
Bilateral cleft lip and palate (BCLP)	0 (0.00%)	0 (0.00%)	0 (0.00%)
Cleft palate (CP)	0 (0.00%)	0 (0.00%)	0 (0.00%)
Cleft palate with unilateral cleft lip (UCLP)	4 (23.52%)	8 (47.06%)	12 (70.59%)
Bilateral cleft lip and palate (BCLP)	0 (0.00%)	1 (5.88%)	1 (5.88%)
Total	8 (47.06%)	9 (52.94%)	17 (100%)

**Table 4:** Association between maternal age and CLPs.

Maternal Age	20–29	30–34	Over 35
Non-syndromic cases	4	2	2
Syndromic cases	6	1	2
Total	10	3	4

**Table 5:** Association between paternal age and CLPs.

Paternal Age	<40	40–49	50–59
Non-syndromic cases	4	3	1
Syndromic cases	7	1	1
Total	11	4	2

## Data Availability

The data presented in this study are available on request from the corresponding author.
